# Development and Validation of a Novel Diagnostic Test for Human Brucellosis Using a Glyco-engineered Antigen Coupled to Magnetic Beads

**DOI:** 10.1371/journal.pntd.0002048

**Published:** 2013-02-14

**Authors:** Andrés E. Ciocchini, Diego A. Rey Serantes, Luciano J. Melli, Jeremy A. Iwashkiw, Bettina Deodato, Jorge Wallach, Mario F. Feldman, Juan E. Ugalde, Diego J. Comerci

**Affiliations:** 1 Instituto de Investigaciones Biotecnológicas Dr. Rodolfo A. Ugalde, Universidad Nacional de San Martín, San Martín, Buenos Aires, Argentina; 2 Alberta Glycomics Centre, Department of Biological Sciences, University of Alberta, Edmonton, Canada; 3 Unidad de Enfermedades Infecciosas, Hospital F.J. Muñiz, Buenos Aires, Argentina; 4 Comisión Nacional de Energía Atómica, Grupo Pecuario, Centro Atómico Ezeiza, Buenos Aires, Argentina; University of California San Diego School of Medicine, United States of America

## Abstract

Brucellosis is a highly contagious zoonosis and still a major human health problem in endemic areas of the world. Although several diagnostic tools are available, most of them are difficult to implement especially in developing countries where complex health facilities are limited. Taking advantage of the identical structure and composition of the *Brucella spp.* and *Yersinia enterocolitica* O:9 *O*-polysaccharide, we explored the application of a recombinant *Y. enterocolitica O:9*-polysaccharide-protein conjugate (OAg-AcrA) as a novel antigen for diagnosis of human brucellosis. We have developed and validated an indirect immunoassay using OAg-AcrA coupled to magnetic beads. OAg-AcrA was produced and purified with high yields in *Y. enterocolitica* O:9 cells co-expressing the oligosaccharyltransferase PglB and the protein acceptor AcrA of *Campylobacter jejuni* without the need for culturing *Brucella*. Expression of PglB and AcrA in *Y. enterocolitica* resulted in the transfer of the host *O*-polysaccharide from its lipid carrier to AcrA. To validate the assay and determine the cutoff values, a receiver-operating characteristic analysis was performed using a panel of characterized serum samples obtained from healthy individuals and patients of different clinical groups. Our results indicate that, using this assay, it is possible to detect infection caused by the three main human brucellosis agents (*B. abortus*, *B. melitensis* and *B. suis*) and select different cutoff points to adjust sensitivity and specificity levels as needed. A cutoff value of 13.20% gave a sensitivity of 100% and a specificity of 98.57%, and a cutoff value of 16.15% resulted in a test sensitivity and specificity of 93.48% and 100%, respectively. The high diagnostic accuracy, low cost, reduced assay time and simplicity of this new glycoconjugate-magnetic beads assay makes it an attractive diagnostic tool for using not only in clinics and brucellosis reference laboratories but also in locations with limited laboratory infrastructure and/or minimally trained community health workers.

## Author Summary

Brucellosis is one of the most widespread zoonoses in the world and still represents a mayor animal and human health problem in many endemic areas. Central for early diagnosis and appropriate treatment of the disease in humans is to have an easy to implement, fast and accurate diagnostic test for the disease. Currently, most of the diagnostic tests for human brucellosis are not of simple implementation in areas where laboratory infrastructure or trained personnel are not available. We present here a novel and simple diagnostic test for human brucellosis using a glyco-engineered antigen coupled to magnetic beads. Our results show that, with this new method, it is possible to detect infection caused by the three main human brucellosis agents with high sensitivity and specificity. We additionally demonstrate that this assay allows the selection of different cutoff points so that the desired operating characteristics of the test in terms of diagnostic sensitivity and specificity can be adjusted according to the needs of the operator. We believe that the high accuracy, low cost and simplicity of this new test makes it an attractive diagnostic tool not only for clinics and brucellosis reference laboratories but also for locations with limited laboratory infrastructure and/or minimally trained community health workers.

## Introduction

Human brucellosis is one of the world's most widespread bacterial zoonoses and, over the past decade, new foci of the disease have emerged. Every year, more than 500,000 new cases are reported globally with annual incidence rates that varies widely (from <2 to >500 per 1,000,000 population) among different regions [1]. The etiological agents of brucellosis are Gram-negative bacteria of the genus *Brucella*. *B. melitensis, B. abortus* and *B. suis* are the three main human brucellosis pathogens whose preferred natural host animals are sheep and goats, cattle, and swine, respectively. The infection is primarily transmitted by consumption of unpasteurized dairy products, direct contact with infected animals (slaughterhouse workers and veterinarians), and handling of cultures and clinical specimens in clinical, research, and production laboratories. Since brucellosis is an important cause of veterinary morbidity and mortality, it causes important economic losses in endemic regions. Additionally, the disease can lead to serious complications in affected patients with an important public health issue. In this regard, when evaluating the economic impact of the disease, this should include not only the cost of treatment and diagnosis, but also the cost in terms of disability-adjusted life years [2], [3].

The wide spectrum of clinical manifestations and lack of pathognomonic symptoms make human brucellosis difficult to clinically diagnose and distinguish from several febrile conditions that often occur in the same areas; therefore, laboratory tests are essential for diagnosing the disease. Even though isolation of *Brucella*, mainly from blood, continues to be the gold standard method for the diagnosis of brucellosis, it presents several drawbacks. The slow growth of *Brucella* in primary cultures delays diagnosis for several days and the sensitivity is often low ranging from 50 to 90% depending on the stage of the disease, previous use of antibiotics, the clinical specimen, and the culture method employed. Furthermore, culturing *Brucella* sp. is hazardous and not all the laboratories have suitable culture facilities [2], [4], [5]. Hence, when the disease cannot be confirmed by culture, serological tests play a major role in the routine diagnosis of brucellosis.

Currently, the diagnostic methods most commonly used for human serological testing are agglutination, complement fixation, and enzyme immunoassay tests [4], [5]. All these assays use as antigen the whole bacteria or bacterial extracts containing high concentrations of the smooth lipopolysaccharide (sLPS) since the humoral immune response to smooth *brucellae* is dominated by antibodies to the *O*-polysaccharide section of *Brucella* sLPS.

In a previous work, we have produced a recombinant glycoconjugate consisting in the *O-*polysaccharide (OAg) fraction of *Yersinia enterocolitica* O:9 LPS covalently linked to the carrier protein AcrA derived from *Campylobacter jejuni* (herein after OAg-AcrA) [6]. The glycoprotein OAg-AcrA was produced and purified using an *in vivo* bacterial glycosylation system that is based on the combination of the LPS biosynthesis pathway and the *N*-glycosylation pathway of *Campylobacter jejuni* [7]. In this system, several glycosyltransferases sequentially add the sugars required for the synthesis of a heptasacharide to the lipid-carrier undecaprenyl pyrophosphate at the cytoplasmic side of the inner membrane. The lipid-linked oligosaccharide is flipped into the periplasm and transferred to the proteins by the oligosaccharyltransferase (OTase) PglB. Because PglB has relaxed glycan specificity, co-expression of *C. jejuni* AcrA and PglB in *Yersinia enterocolitica* O:9 resulted in glycosylation of AcrA with the host OAg, which is also assembled onto the undecaprenyl pyrophosphate lipid carrier by a dedicated biosynthetic machinery consisting of a set of sugar modifying enzymes, glycosyltransferases, and membrane transporters [6]. This machinery is common to *Y. enterocolitica* O:9 and *B. abortus*. Mass spectrometry analysis of the resulting glycoprotein demonstrated the transfer to AcrA of a homopolymer of *N*-formylperosamine identical to the structure of *Y. enterocolitica* O:9 and *B. abortus O*-polysaccharide [6]. The saccharide moiety, but not the carrier protein AcrA, was recognized by monoclonal antibodies against *Y. enterocolitica* O:9 and *B. abortus O*-polysaccharides, as well as by bovine sera obtained from animals infected with a virulent strain of *B. abortus*. Furthermore, using OAg-AcrA as antigen we were able to clearly differentiate *B. abortus*-infected from non-infected animals demonstrating the potential of this glycoconjugate to develop new diagnostic tools for brucellosis [6].

In the present work, we have developed and validated a magnetic beads-based immunoassay using the recombinant *O:9*-polysaccharide-protein conjugate OAg-AcrA as a novel antigen for the diagnosis of human brucellosis.

## Materials and Methods

### Ethics statement

In this study, we have analyzed a previously characterized sera collection provided by the Brucellosis Division from the Hospital de Enfermedades Infecciosas Francisco Javier Muñiz (Buenos Aires, Argentina). These samples were obtained from patients who were admitted to the hospital between the years 2008–2011 and the data were analyzed anonymously. Blood donors' samples were obtained under informed consent and provided by Fundación Hemocentro Buenos Aires (Buenos Aires, Argentina).

### Production and purification of the O:9-polysaccharide-protein conjugate (OAg-AcrA)

Production and purification of the OAg-AcrA glycoconjugate was performed as previously described [6]. *Yersinia enterocolitica* O:9 wild type strain transformed with the plasmids pMAF10 (encoding the *Campylobacter jejuni* OTase PglB) and pMH5 (encoding the *C. jejuni* carrier protein AcrA fused to an histidine tag) was grown overnight at 37°C. Cultures were re-inoculated at 1/100 dilution into fresh LB media and grown at 37°C for 2.5 h (OD_600_∼0.5) and PglB_Cj_ expression was induced with addition of arabinose to a final concentration of 0.2% (w/v). Four hours after induction at 37°C, PglB_Cj_ was re-induced by a second addition of arabinose to maximize glycosylation of AcrA. Cells were harvested by centrifugation after a 20 h induction period and periplasmic extracts were prepared by lysozyme treatment as described elsewhere [7]. Subsequently, the periplasmic fraction was equilibrated with 1/9 vol 10×loading buffer (0.1 M imidazole, 3 M NaCl, 0.2 M Tris-HCl pH 8.0) and subjected to Ni^2+^ affinity chromatography. The column was equilibrated with 10 column volumes of 1× loading buffer and loaded on a HisTrap HP column (Amersham Pharmacia Biosciences) at a flow rate of 1 ml/min. The column was washed with 25 column volumes of wash buffer (0.02 M imidazole, 0.3 M NaCl, 0.02 M Tris-HCl pH 8.0), and eluted from the column with the elution buffer (0.25 M imidazole, 0.3 M NaCl, 0.02 M Tris-HCl pH 8.0).

### Glycoconjugate magnetic beads-based immunoassay development and optimization

Superparamagnetic COOH-modified microbeads (Bangs Laboratories) were activated in one step with EDAC [1-ethyl-3-(3-dimethyl-aminopropyl) carbodiimide hydrochloride] and NHS [N-hydroxy succinimide] in 100 mM MES (2-[N-Morpholino] ethanesulfonic acid) pH 5.5 buffer. Activated beads were washed with 0.01 M phosphate-buffered saline pH 7.2 (PBS) and then incubated with different amounts of OAg-AcrA. Resulting functionalized magnetic beads were washed with quenching buffer (35 mM glycine, 1% gelatin from cold water fish skin) and incubated overnight at 4°C with the same buffer. Finally, OAg-AcrA-beads were washed with storage buffer (1% gelatin from cold water fish skin, 2.25% Tween 20, 0.01% sodium azide), resuspended in the same buffer and stored at 4°C until use.

To perform the assay, OAg-AcrA-functionalized microbeads were incubated with human serum samples, washed two times with 0.01 M phosphate-buffered saline pH 7.2 containing 0.2% Tween 20 (PBST) and bound antibodies were detected incubating the beads with Cy5-conjugated goat anti human IgM/G antibodies (Jackson ImmunoResearch Laboratories). After washing twice with PBST, fluorescence was determined using a plate fluorometer (DTX 880 Multimode Detector, BeckmanCoulter). All washes were done using a magnetic rack.

In order to determine the optimal antigen concentration, sample and antibody dilutions and incubation times, a checkerboard titration analysis was carried out using high-, medium-, low-positive and negative sera, and checking for strong versus low background. Based on these analyses the optimal amount of antigen was 2 µg per reaction, the optimal serum sample dilution was at 1/100 and the incubation times with the sample and the conjugate was reduced to 5 minutes at room temperature. These parameters were used to test all the samples.

### Human serum samples

A total of 488 serum samples of healthy individuals and patients belonging to different clinical groups were analyzed (see Figure S1 and Table S3 in the supplemental material).

- Culture-positive brucellosis patients: patients with positive blood cultures for *Brucella abortus, Brucella melitensis* or *Brucella suis* (52 serum samples of 25 patients).
- Culture-negative brucellosis patients: culture-negative brucellosis patients with clinical diagnosis of brucellosis and serologically-positive by Rose Bengal Test (RBT), Serum Agglutination Test (SAT; Huddleson), Tube Agglutination Test (TAT; Wright), TAT-2ME (2-mercaptoethanol), Competitive ELISA (CELISA) and/or Complement Fixation Test (CFT) (86 samples of 48 patients). The CELISA was performed as indicated in [8].
- Blood donors: healthy individuals with no epidemiological evidence of brucellosis and negative agglutination tests (240 serum samples).
- Patients with febrile syndrome: patients with febrile illness whose serum samples were negative by RBT, SAT, TAT, TAT-2ME, CELISA and CFT (34 serum samples).
- Healthy individuals occupational exposed to the pathogen: research, clinical and production laboratory workers, and cattle ranchers (30 serum samples).
- Patients with other bacterial infections or with autoimmune pathologies: patients infected with *Escherichia coli* O157:H7 (14 serum samples), *Mycobacterium tuberculosis* (9 serum samples), *Klebsiella pneumoniae* (3 serum samples), *Acinetobacter baumannii* (4 serum samples), *Pseudomonas aeruginosa* (1 serum sample), or *Streptococcus pneumoniae* (1 serum sample), or with autoimmune pathologies such as rheumatoid arthritis, systemic lupus erythematosus or autoimmune vasculitis (14 serum samples).

### Western blotting

Non-glycosylated and glycosylated AcrA were subjected to 10% sodium dodecyl sulfate-polyacrylamide gel electrophoresis (SDS-PAGE) [9], and transferred to a nitrocellulose membrane with a semi-dry membrane transfer. Immunoblotting was performed as previously described [10] with human serum samples, and mouse anti-*Brucella O*-polysaccharide (M84) [11] and anti-histidine tag (Qiagen) monoclonal antibodies. Bound antibodies were visualized using horseradish peroxidase-conjugated goat anti-human IgG (Sigma) secondary antibodies and enhanced chemiluminescence (Supersignal West Pico chemiluminescent substrate detection reagents, Pierce Chemical Co.), according to the manufacturer's instructions.

### Data analysis

Results were expressed as percentage of reactivity of the mean Fluorescence Units (FU) of the positive control serum included in each assay run. Percentage of reactivity was calculated as follows: % of reactivity = (FU of the test sample/mean FU of the positive control)×100.

Dot, receiver-operating characteristic (ROC) and Spearman's correlation analyses were performed using the GraphPad Prism software (version 5.01 for Windows, San Diego California USA, [http://www.graphpad.com]).

## Results

### OAg-AcrA glycoconjugate as a novel antigen for the diagnosis of human brucellosis

**Figure 1:**
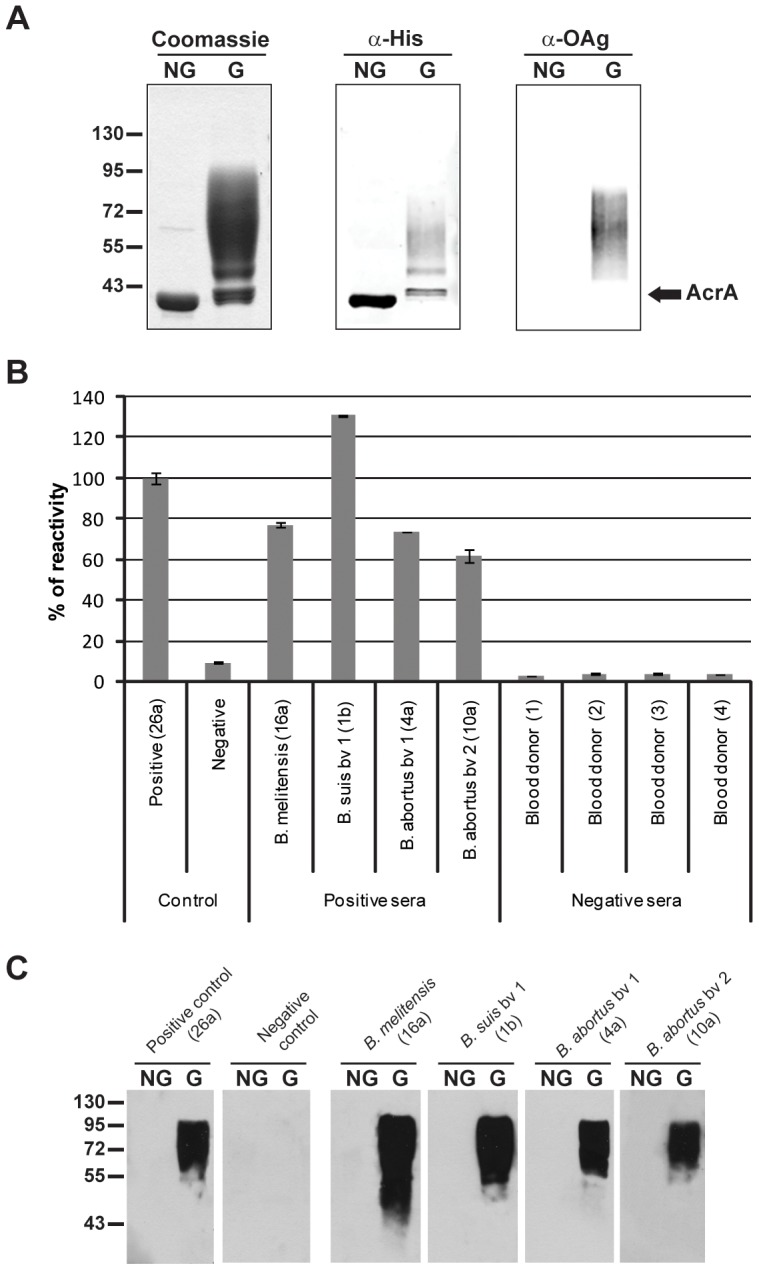
OAg-AcrA glycoconjugate as an antigen for the diagnosis of human brucellosis. A) 10% SDS-PAGE analysis of purified non-glycosylated (NG) and glycosylated AcrA (G) by Coomassie brilliant blue and immunoblot using anti-histidine tag and anti-*Brucella O*-antigen monoclonal antibodies. B) Glycoconjugate magnetic beads-based immunoassay for detection of antibodies against the *O*-polysaccharide fraction of lipopolysaccharide (LPS). Magnetic beads coated with OAg-AcrA were incubated with the indicated human serum samples. Bound antibodies were detected using Cy5-conjugated goat anti human IgM/G antibodies.Positive and negative controls; human sera included as positive and negative controls in each assay run. The figures in parenthesis correspond to the identification of the samples according to Tables S1 and S2. Results are expressed as percentage of reactivity of the positive control serum. The bar graph data represents the means and standard deviation for two separate determinations. C) Western blot analysis of the same human serum samples.

An indirect immunoassay based on the detection of anti *O*-polysaccharide IgG/M antibodies in serum samples was developed using as antigen the recombinant *O:9*-polysaccharide-protein conjugate (OAg-AcrA). The glycoprotein OAg-AcrA was devised and produced using an *in vivo* bacterial glycosylation system, as previously described [6], purified from periplasmic extracts by affinity chromatography and characterized by Western blot analysis. As shown in Figure 1A, the glycosylated form of AcrA was recognized by the anti-histidine tag monoclonal antibody (AcrA was expressed as an histidine tag fusion protein) and an anti-*Brucella O*-antigen monoclonal antibody, while the non-glycosylated form was only detected by the anti-histidine tag antibody.

As a proof of concept and to demonstrate the feasibility of the use of OAg-AcrA antigen for the diagnosis of human brucellosis, we analyzed serum samples obtained from patients with culture-confirmed brucellosis by *B. abortus* (bv1 and bv2), *B. suis* (bv1) and *B. melitensis* as well as from healthy blood donors (Figure 1B). To perform the assay, super-paramagnetic beads functionalized with the antigen were incubated with the samples for 5 min at RT and washes were performed using a magnetic rack without the need of centrifugation. Then, magnetic beads were incubated with Cy5-conjugated anti-human IgM/G antibodies (5 min at RT), washed, and detection was performed directly by reading the fluorescence without the need to add an additional substrate. The obtained results demonstrate that using the recombinant glycoconjugate as antigen it is possible to discriminate between infected patients and non-infected individuals. Furthermore, to evaluate the specificity of the reaction, the same sera were analyzed by Western blot against the non-glycosylated and glycosylated forms of AcrA (Figure 1C). Reactivity against glycosylated AcrA was observed with the serum samples obtained from infected patients but not with the samples of healthy blood donors, and none of the samples were reactive against the non-glycosylated form of AcrA. These results indicate that the detected antibody response is specifically directed towards the *O*-polysaccharide moiety of the glycoconjugate.

Taken together these results demonstrate the potential of this glyco-engineered antigen-based assay for the diagnosis of human brucellosis caused by the three main human brucellosis pathogens.

### Glycoconjugate magnetic beads-based assay validation

**Figure 2:**
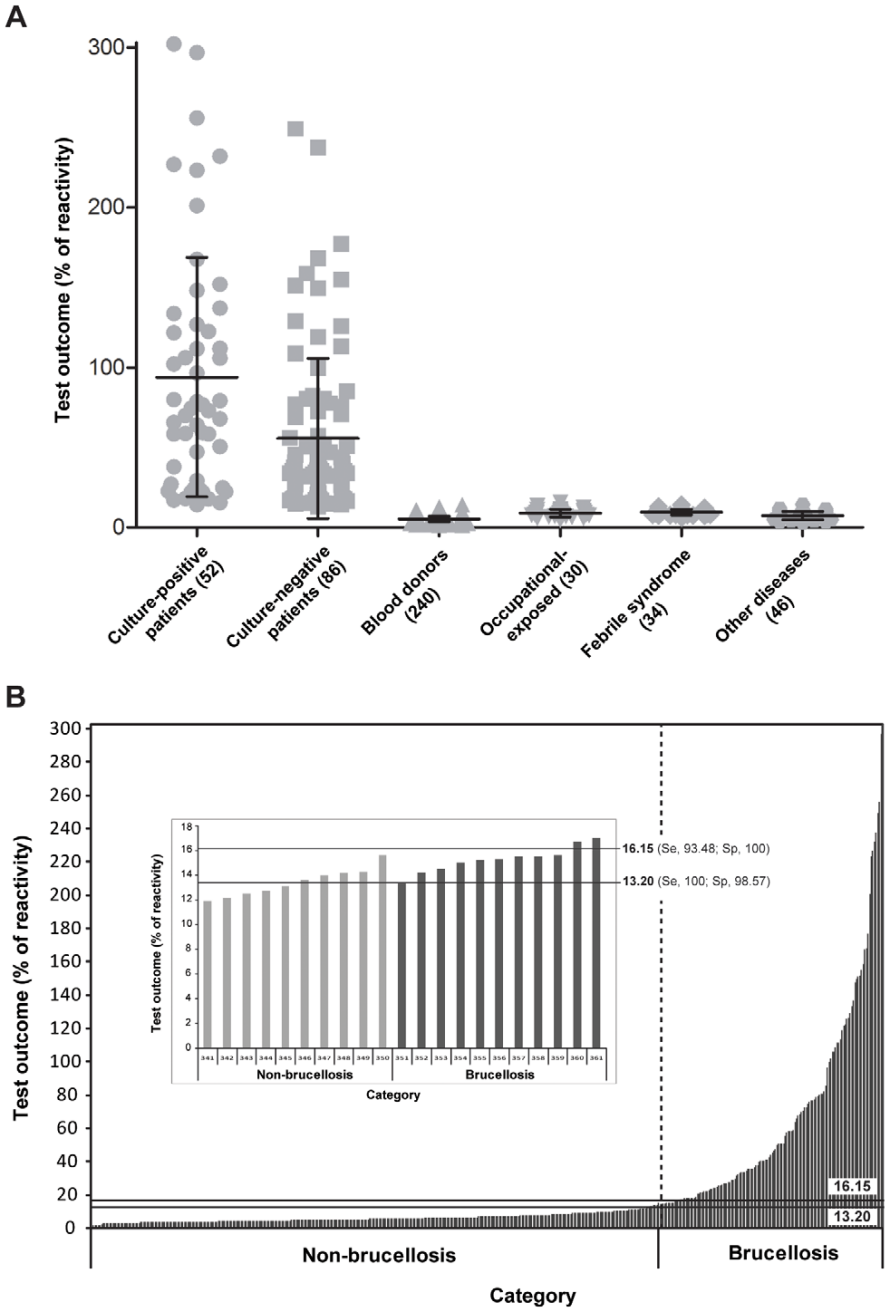
Glycoconjugate-magnetic beads assay analysis of serum samples obtained from healthy individuals and patients of different clinical groups. A) Dotplot of the glycoconjugate-magnetic beads assay results. Serum samples of blood culture-positive patients (52 sera), culture-negative/serologically-positive patients with clinical diagnosis of brucellosis (86 sera), blood donors (240 sera), healthy individuals occupational-exposed to the pathogen (30 sera), patients with febrile syndrome (34 sera) and patients with other diseases (46 sera) were tested as indicated in Materials and Methods. The mean and standard deviation for each group are indicated. B) Analysis of glycoconjugate-magnetic beads assay results classifying the samples into two categories. Non-brucellosis category includes samples obtained from blood donors, healthy individuals occupational-exposed to the pathogen, patients with febrile syndrome and patients with other diseases. Brucellosis category includes samples of blood culture-positive and culture-negative/serologically-positive patients with clinical diagnosis of brucellosis. For each category reactivity values are arrange in increasing order. The horizontal lines indicate the cutoff values calculated by ROC analysis for which maximal sensitivity (Se) or specificity (Sp) is achieved. The inset shows a zoom of the graph part displaying the limit between categories.

To validate the assay, a total of 488 serum samples obtained from healthy individuals and patients of different clinical groups were tested as indicated in Materials and Methods. The reactivity values of these samples were outlined in a dotplot diagram as shown in Figure 2A. The results indicate that with this assay it is possible to clearly discriminate between culture-positive patients (mean value ± SD, 93.90±74.78%) and culture-negative/serologically-positive patients with clinical diagnosis of brucellosis (55.66±50.06) from blood donors (5.19±1.80), healthy individuals occupational exposed to the pathogen (8.91±2.40), patients with febrile syndrome (9.54±1.76) and patients with other infectious or autoimmune diseases (7,25±2.53).

A further analysis of the results was performed classifying the samples into two categories (Figure 2B). The non-brucellosis category includes samples obtained from blood donors, healthy individuals occupational exposed to the pathogen, patients with febrile syndrome and patients with other diseases. The brucellosis category includes samples of patients with culture-confirmed brucellosis and culture-negative but serologically positive patients with clinical diagnosis of brucellosis. For each category reactivity values were arranged in increasing order and its distribution showed a minimal overlap between categories (see inset in Figure 2B) indicating that it is possible to select different cutoff points so that the desired operating characteristics of the test in terms of diagnostic sensitivity and specificity can be adjusted (see below).

To evaluate the diagnostic performance of the assay and determine the cutoff values that concurrently optimize sensitivity and specificity, a receiver-operating characteristic (ROC) analysis was performed. The underlying assumption of ROC analysis is that a diagnostic variable (e.g. glycoconjugate-magnetic beads assay values) is used to discriminate between two mutually exclusive states of tested individuals. At first, ROC analysis was performed considering as negative controls only the serum samples obtained from healthy blood-donors (240 sera). Serum samples obtained from patients with culture-confirmed brucellosis and culture-negative/serologically-positive patients with clinical diagnosis of brucellosis (138 sera) were used as positive controls. Based on this analysis, the area under the ROC curve (AUC) for the test was 1.000 (95% CI, 0.9999 to 1.000) and the optimum cutoff value was 13.00% which resulted in a diagnostic sensitivity of 100% (95% CI, 97.36 to 100) and specificity of 99.58% (95% CI, 97.70 to 99.99) with only one false positive. When a cutoff value of 14.18% was selected the sensitivity fell to 99.28% (95% CI, 96.03 to 99.98), with one false negative, and the specificity increased to 100% (95% CI, 98.47 to 100).

**Figure 3:**
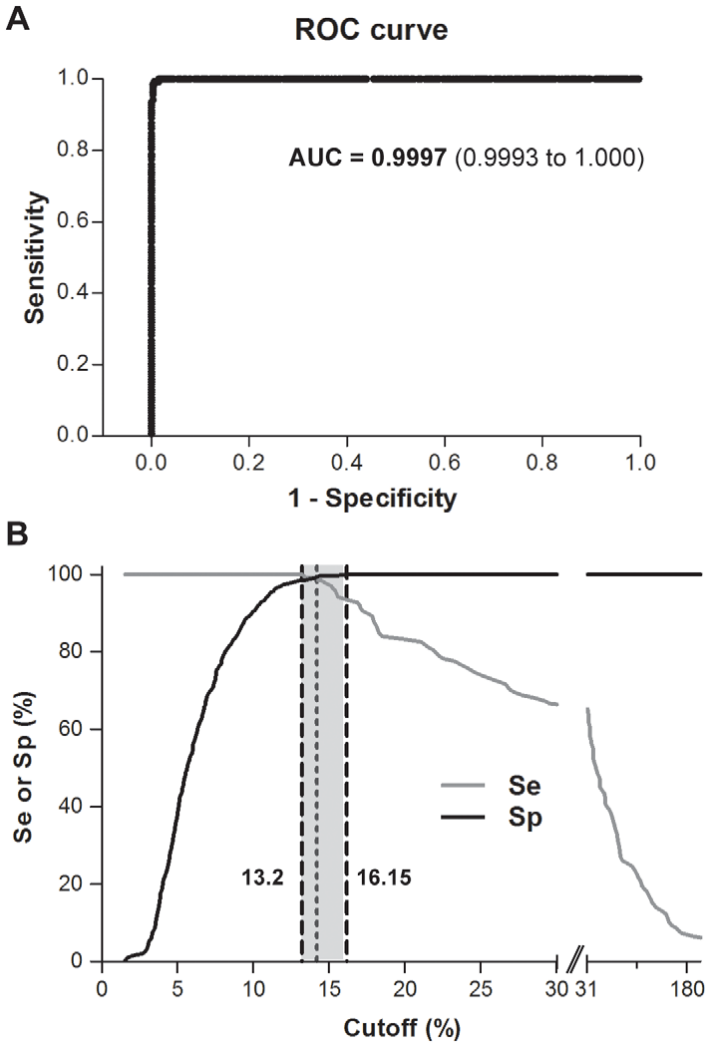
Receiver-operating characteristic (ROC) analysis of glycoconjugate-magnetic beads assay results. A) ROC plot. The analysis was carried out considering as positive controls sera of patients with culture-confirmed brucellosis and culture-negative/serologically-positive patients with clinical diagnosis of brucellosis (138 sera), and as negative controls serum samples from blood-donors, healthy individuals occupational-exposed to the pathogen, patients with febrile syndrome and patients with other diseases (350 sera). AUC, area under the ROC curve. Values in parentheses indicate the 95% confidence interval. B) Plot of the diagnostic sensitivity (Se) and specificity (Sp) of the assay as a function of the cutoff value. The dot vertical line indicates the cutoff value that concurrently optimizes Se and Sp (cutoff = 14.18%, Se = 99.28% and Sp = 99.43%). The dash vertical lines indicate the cutoff values for which maximal Se or Sp is achieved (cutoff = 13.20%, Se = 100% and Sp = 98.57%; cutoff = 16.15%, Se = 93.48% and Sp = 100%).

However, considering as negative controls only the samples of healthy blood-donors possibly overestimates the test specificity because patients who may have cross-reacting antibodies, such as those with clinical suspicion of brucellosis, exposed to the pathogen or with other diseases, are not being considered in the analysis. Thus, a second ROC analysis was carried out including as negative controls the serum samples obtained from all the clinical groups previously included in the non-brucellosis category (350 sera, see Figure 2B). In this case, the AUC for the test was 0.9997 (95% CI, 0.9993 to 1.000) indicating that the glycoconjugate-magnetic beads assay is highly accurate for the diagnosis of human brucellosis (Figure 3A). Furthermore, a plot of sensitivity and specificity as a function of the cutoff values allowed us to determine the cutoff value (14.18%) that maximizes sensitivity and specificity and select two different cutoff values (13.20 and 16.15%) for definition of intermediate test results (test results that are considered non-negative and non-positive) (Figure 3B). As shown in Table 1, the optimum cutoff value of 14.18% resulted in a diagnostic sensitivity and specificity of 99.28% (95% CI, 96.03 to 99.98) and 99.43 (95% CI, 97.95 to 99.93), respectively, with one false-negative and two false-positive results. The cutoff value of 13.20% resulted in a diagnostic sensitivity of 100% (95% CI, 97.36 to 100), a diagnostic specificity of 98.57% (95% CI,96.70 to 99.53), with 5 false positives, and a negative predictive value of 100%. A cutoff value of 16.15% resulted in a test sensitivity of 93.48% (95% CI, 87.98 to 96.97) with 9 false negatives, a test specificity of 100% (95% CI, 98.91 to 100) and a positive predictive value of 100%.

**Table 1:** Sensitivity, specificity, performance index, and positive and negative predictive values of the test calculated for different cutoff values^a^.

Cutoff (%)	Se (%)^b^	Sp (%)^b^	PI^c^	PPV (%)^d^	NPV (%)^d^	TP	TN	FP	FN
>13.20	**100** e (97.36–100)	98.57 (96.70–99.53)	198.57	96.50	**100**	138	345	5	0
>13.45	99.28 (96.03–99.98)	98.57 (96.70–99.53)	197.85	96.48	99.71	137	345	5	1
>13.80	99.28 (96.03–99.98)	98.86 (97.10–99.69)	198.14	97.16	99.71	137	346	4	1
>14.08	99.28 (96.03–99.98)	99.14 (97.52–99.82)	198.42	97.86	99.71	137	347	3	1
>14.18	99.28 (96.03–99.98)	99.43 (97.95–99.93)	**198.71**	98.56	99.71	137	348	2	1
>14.24	98.55 (94.86–99.82)	99.43 (97.95–99.93)	197.98	98.55	99.43	136	348	2	2
>14.39	98.55 (94.86–99.82)	99.71 (98.42–99.99)	198.26	99.27	99.43	136	349	1	2
>14.75	97.83 (93.78–99.55)	99.71 (98.42–99.99)	197.54	99.26	99.15	135	349	1	3
>15.10	97.10 (92.74–99.20)	99.71 (98.42–99.99)	196.81	99.26	98.87	134	349	1	4
>15.25	96.38 (91.75–98.81)	99.71 (98.42–99.99)	196.09	99.25	98.59	133	349	1	5
>15.40	95.65 (90.78–98.39)	99.71 (98.42–99.99)	195.36	99.25	98.31	132	349	1	6
>15.55	94.20 (88.90–97.46)	99.71 (98.42–99.99)	193.91	99.24	97.76	130	349	1	8
>16.15	93.48 (87.98–96.97)	**100** (98.95–100)	193.48	**100**	97.49	129	350	0	9

a The analysis was performed using 138 sera as positive-controls and 350 sera as negative-controls.

b Se, sensitivity (TP/TP+FN)×100; Sp, specificity (TN/TN+FP)×100. Values in parentheses indicate the 95% confidence interval. TP, true positives; TN, true negatives; FP, false positives; FN, false negatives.

c PI, performance index calculated as the sum of the sensitivity and specificity values.

d PPV, positive predictive value (TP/TP+FP)×100; NPV, negative predictive value (TN/TN+FN)×100.

e Maximum values for Se, Sp, PI, PPV and NPV are indicated in bold.

Fifty-two samples of 25 patients with blood culture-confirmed brucellosis were tested. In 6 patients the isolated strain was *B. suis*, in 8 cases *B. abortus* and in 11 cases *B. melitensis*. In one patient, who had a right knee bursitis, the bacterium was isolated from the synovial fluid. Interestingly, the serum and synovial fluid samples were positive by the glycoconjugate-beads assay suggesting that our assay could be used to diagnose the disease using different types of samples. In this group the sensitivity of the glycoconjugate-beads assay (cutoff >13.20%) was 100% demonstrating that this assay can be used to diagnose brucellosis caused by the three main human brucellosis pathogens. When considering all the positive samples obtained from culture-positive and culture-negative brucellosis patients the diagnostic sensitivity of the glycoconjugate-beads assay and CELISA was significantly higher than the sensitivity of RBT, SAT, TAT and CFT (Table 2).

**Table 2:** Sensitivity values of the different serological tests^a^.

Number of samples	TEST (cutoff)	TP	FN	Se (%)
138	RBT	84	54	60.87
138	SAT (≥100)	70	68	50.72
138	SAT (≥25)	121	17	87.68
138	TAT(≥100)	64	74	46.37
138	TAT(≥25)	123	15	89.13
127	CELISA (>28%I)^b^	125	2	98.43
125	CFT (≥5)	95	30	76.00
138	GBA^c^ (>13.20%)	138	0	100
138	GBA^c^ (>16.15%)	129	9	93.48

a The analysis was performed considering as positive reference controls sera obtained from patients with culture-confirmed brucellosis (52 sera) and from culture-negative/serologically-positive patients with clinical diagnosis of brucellosis (86 sera).

b %I, percentage of inhibition.

c GBA, glycoconjugate-beads assay.

TP, true positives; FN, false negatives; Se, sensitivity (TP/TP+FN)×100.

Finally, we analyzed samples of nine brucellosis patients who received antibiotic therapy. The serological follow-up performed with serial serum samples obtained at admission and 1, 2, or more months after treatment showed that the glycoconjugate-beads assay values correlated very well with CELISA and CFT results (Table 3, and Tables S1 and S2 in the supplemental material). When a Spearman's correlation analysis was performed considering all the positive reference samples (138 sera), the correlation coefficient between the glycoconjugate-beads assay and CELISA was 0.7771 (P<0.0001, 95% CI 0.6945 to 0.8394), between our assay and CFT was 0.8155 (P<0.0001, 95% CI 0.7441 to 0.8684), while the correlation between CELISA and CFT fell to 0.7254 (P<0.0001, 95% CI 0.6264 to 0.8014).

**Table 3:** Serological follow-up of patients treated with antibiotic therapy.

Patient N°	Samples^a^	Blood Culture	Glycoconjugate-beads assay (%)^b^	CELISA (%I)^c^	FC^d^
28	a(0)	Negative	155.1	81	320
	b(2)		85.6	66	80
	c(4)		50.7	48	40
	d(7)		26.8	46	40
36	a(0)	Negative	113.4	74	160
	b(7)		41.5	56	20
	c(11)		51.1	45	5
	d(16)		17.9	42	5
	e(19)		40.2	45	5
	f(23)		35.6	40	5
1	a(0)	*B. suis* bv 1	79.3	76	1280
	b(2)		133.6	76	640
	c(5)		255.9	94	1280
	d(9)		296.9	91	640
	e(13)		223.3	92	320
	f(17)		201.1	81	160
	g(20)		137.0	55	80
	h(23)		151.9	60	80
	i(26)		111.8	73	40
	j(29)		122.4	75	40
3	a(0)	*B. suis* bv 1	105.9	63	320
	b(13)		302.3	91	160
	c(17)		226.9	89	160
	d(20)		232.1	85	80
	e(30)		111.7	72	20
	f(32)		126.8	71	20
6	a(0)	*B. abortus* bv 1	102.2	64	320
	b(3)		65.6	61	80
	c(8)		58.5	48	40
7	a(0)	*B. suis* bv 1	121.6	64	160
	b(2)		167.4	74	320
	c(6)		74.6	52	40
10	a(0)	*B. abortus* bv 2	69.9	65	10
	b(5)		27.0	45	5
	c(10)		22.3	43	NEG
	d(12)		15.5	30	NEG
37	a(0)	Negative	129.2	68	80
	b(2)		77.1	48	80
	c(3)		82.6	52	40
	d(6)		72.8	62	20
	e(10)		47.0	60	20
	f(32)		24.5	49	10
39	a(0)	Negative	158.8	88	160
	b(3)		237.5	92	320
	c(4)		249.3	93	640
	d(6)		151.2	92	320
	e(8)		125.9	91	80
	f(10)		80.7	88	80
	g(13)		68.9	82	20
	h(20)		41.1	75	5
	i(31)		25.4	65	5

a The letters indicate consecutive serum samples. The numbers in parenthesis indicate the months at which the samples were taken after admission.

b Results are expressed as percentage of reactivity of the control positive serum. Cutoff >13.2%.

c Cutoff >28%I.

d Results are shown as titers. Cutoff ≥5.

## Discussion

Timely and accurate diagnosis of human brucellosis continues to challenge clinicians due to its non-specific clinical features, the slow growth rate of *Brucella* spp. in blood cultures, and the complexity of its serodiagnosis [2]. Since the humoral immune response to “smooth” *brucellae* is dominated by antibodies to the *O*-polysaccharide section of *Brucella* sLPS and because *Yersinia enterocolitica* O:9 and *Brucella abortus* share an almost identical *O*-polysaccharide structure, we explored the application of the recombinant *Y. enterocolitica O:9*-polysaccharide-protein conjugate (OAg-AcrA) in the diagnostics of human brucellosis.

In the present work we described the development, optimization and validation of an indirect immunoassay to detect antibodies against “smooth” *Brucella* spp. using the recombinant glycoconjugate OAg-AcrA antigen coupled to magnetic beads. The assay was validated using a panel of characterized serum samples obtained from healthy individuals and patients of different clinical groups including not only patients with brucellosis but also patients with febrile syndrome and symptoms compatible with brucellosis as well as patients with other infections or autoimmune diseases, who may have cross-reacting antibodies. The obtained results allowed us to validate the assay and select different cutoff values taking into account the actual epidemiological situation of the disease. We demonstrated that using the glycoconjugate-beads assay it is possible to clearly discriminate between infected and non-infected individuals and that this assay is efficient to detect infections caused by the three main human brucellosis pathogens: *B. abortus*, *B. melitensis* and *B. suis*. We also confirmed by immunoblot that the detected antibody response in positive serum samples is specifically directed towards the *O*-polysaccharide moiety of the glycoconjugate. To evaluate the performance of the assay a receiver-operating characteristic (ROC) analysis was performed. ROC analysis provides a precise and valid measure of diagnostic accuracy and assesses the diagnostic performance of the system in terms of the true-positive rate (sensitivity) and false-positive rate (1-specificity) for each possible cutoff value of the test [12], [13]. The area under the ROC curve (AUC) is a global summary statistic of diagnostic accuracy and one could distinguish between non-informative (AUC = 0.5), less accurate (0.5<AUC<0.7), moderately accurate (0.7<AUC<0.9), highly accurate (0.9<AUC<1) and perfect tests (AUC = 1) [13]. For our assay, the AUC was 0.9997 indicating that the glycoconjugate-beads assay is a highly accurate test to diagnose human brucellosis. The cutoff value that concurrently optimizes sensitivity and specificity was determined to be 14.18%, which resulted in a diagnostic sensitivity and specificity of 99.28% and 99.43%, respectively. A cutoff value of 13.20% gave a diagnostic sensitivity of 100% and a diagnostic specificity of 98.571%, and a cutoff value of 16.15% resulted in a test sensitivity of 93.48% and a test specificity of 100%. Based on this analysis, we propose the following criteria for diagnosing human brucellosis; samples with reactivity values less or equal than 13.20% are considered non-reactive, samples with reactivity values higher than 16.15% are considered reactive while samples with values between 13.20 and 16.15% are indeterminate (see Table 1). In this latter case, the result should be confirmed repeating the test two or three weeks later or using another diagnostic method. Considering a cutoff value of 13.20% the diagnostic sensitivity of the glycoconjugate-beads assay was similar to that of CELISA but significantly higher than the sensitivity of the other serological tests considered in this study; furthermore, our test results correlated very well with CELISA and CFT results.

OAg-AcrA was produced in *Y. enterocolitica* O:9 cells co-expressing the *N*-linked protein glycosylation OTase PglB and the protein acceptor AcrA of *Campylobacter jejuni* [6]. Introduction of PglB and AcrA in *Y. enterocolitica* O:9 resulted in the transfer of the *N*-formylperosamine *O*-polysaccharide from its lipid carrier to AcrA and the resulting glycoprotein was purified from the periplasm of the bacteria by affinity chromatography. Most of the diagnostic methods currently used for human serological testing use as antigen whole “smooth” *Brucella* cells or bacterial extracts containing high concentrations of sLPS [4]. In these cases, level-3 biosafety facilities (BSL-3) are required for culturing *Brucella* and production of the antigens. Instead, large quantities of the recombinant glycoconjugate OAg-AcrA antigen can be produced and simply purified from fermentations of *Y. enterocolitica* O:9 (*Y. enterocolitica* O:9 is a biosafety level-2 organism that is easily manipulated and cultured) without the need for culturing *Brucella*, which results in reduced costs and a safer production process. This is relevant considering that brucellosis constitutes one of the most common infections acquired in antigen/vaccine-manufacturing plants and in research and clinical diagnostic laboratories [14], [15], [16], [17]. Furthermore, the glycoengineering technology used in this study to produce the antigen eliminates the need to purify LPS and no chemical treatments are required for isolation of the *O*-polysaccharide from LPS. Finally, no chemical crosslinking of the carbohydrate to the protein is required allowing to produce conjugates with a defined and reproducible sugar pattern since the glycosylation process and the length of the *O*-polysaccharide are controlled *in vivo*. Therefore, this system may enable the production of homogeneous and standardized batches of antigen which may have, in the future, important implications for the diagnosis of brucellosis because until now there is no standardized reference antigen, and it is well known that the source of the antigen used can greatly influence test results.

*B. abortus O*-polysaccharide contains both A (α-1,2-linked homopolymer of *N*-formylperosamine) and M (pentasaccharide with four α-1,2 and one α-1,3-linked polymers of the same sugar) epitopes. *Y. enterocolitica* O:9 *O*-polysaccharide is comprised solely of α-1,2-linked *N*-formylperosamine, *B. abortus* has ∼98% A epitope, *B. suis* has a unique 1∶7 ratio of α1,3- α1,2 linked polymer, whereas *B. melitensis* has only the M epitope of the pentasaccharide repeating unit [18], [19], [20]. These *O*-polysaccharide structural similarities may explain why the OAg-AcrA antigen was reactive towards serum samples obtained from patients with culture-confirmed brucellosis by *B. abortus*, *B. suis* or *B. melitensis*. On the other hand, it is well known that the structural similarity between the *O*-chains of *Yersinia enterocolitica* O:9, smooth *Brucella* spp. and various other clinically relevant bacteria, such us *Salmonella urbana* group N, *Vibrio cholerae*, *Francisella tularensis*, *Escherichia coli* O:157 and *Stenotrophomonas maltophilia*, could lead to cross-reactivity increasing the possibility of false-positive results [21]. In this regard, 14 serum samples of patients with Uremic Hemolytic Syndrome caused by *Escherichia coli* O157:H7 (see the group of patients with other diseases) were analyzed and no cross-reaction was observed. However, due to the possibility of cross-reactions with the bacteria mentioned above, not only the serological tests results but also the clinical and epidemiological context must be considered to confirm the diagnosis. Finally, the whole bacteria and most of the LPS-based assays currently used to diagnose brucellosis not only suffers from false-positive results due to these *O*-chains structural similarities but also due to the presence of cross-reactive antibodies against the common core and lipid A antigens. Instead, the glycoconjugate-beads assay may be more specific than these assays since it uses as antigen only the *O*-polysaccharide fraction of the LPS.

Laboratory testing is essential for diagnosing brucellosis and access to appropriate diagnostic tools is a critical component for early diagnosis and patient management. However, most currently available diagnostic tests may be difficult to implement in resource-poor countries or regions where brucellosis is endemic. An exception are the Rose Bengal Test [22] and the immunochromatographic brucella IgM/IgG Lateral Flow Assay [23] which are simple and rapid methods proposed to be used as POC tests, albeit the fact the interpretation of RBT results are largely subjective [2], [4]. In addition, and especially in developing countries, not all the patients have access to medical services and the local health-care centers may have limited infrastructure and staff might not be able to make or confirm the diagnosis. Presently, 72% of the population in Africa, 42% in Asia and 10% in America only have access to health-care settings with minimal infrastructure or no infrastructure at all [24], [25]. Due to the high diagnostic accuracy, the low cost in terms of production of the antigen, reduced assay time (no more than 15 min), simplicity (no centrifugation and agitation needed and no additional substrate required for detection by fluorescence reading) as well as the availability of portable fluorescence readers, the glycoconjugate-magnetic beads assay may have a great potential as a Point-Of-Care (POC) test for human brucellosis. Therefore, the new glycoconjugate-magnetic beads assay could be a useful diagnostic tool not only for brucellosis reference laboratories or clinics but also for locations with limited laboratory infrastructure and/or minimally trained community health workers. Future work will be needed to validate the assay in regions or countries with lower or higher incidence of brucellosis than in Argentina.

Here, we have demonstrated that the glycoconjugate-beads assay has an excellent performance for the diagnosis of human brucellosis and have validated for the first time a bacterial recombinant glycoprotein as an antigen for the diagnosis of an infectious disease. Further work will be required to validate the recombinant glycoconjugate antigen OAg-AcrA for the diagnosis of animal brucellosis caused by “smooth” *Brucella* spp. strains, as well as to exploit the bacterial glyco-engineering technology to design and produce a panel of glycoconjugates for diagnostics of other relevant human and veterinary infectious diseases.

## Supporting Information

Figure S1 **STARD flow diagram indicating the patients and healthy individuals included in the study.** n_p_, number of patients; n_s_, number of samples.
(DOCX)

Table S1 **Diagnostic laboratory findings in culture-positive brucellosis patients.**
(DOCX)

Table S2 **Serological results in culture-negative, serologically positive brucellosis patients with clinical diagnosis of brucellosis.**
(DOCX)

Table S3 **STARD table checklist.**
(DOC)
