# Supplementary material for: Development and Validation of a Novel Diagnostic Test for Human Brucellosis Using a Glyco-engineered Antigen Coupled to Magnetic Beads
Source: PLoS Negl Trop Dis. 2013 Feb 14;7(2):e2048. doi: 10.1371/journal.pntd.0002048 (PMC3573069; doi:10.1371/journal.pntd.0002048)
Supplement: Figure S1 — STARD flow diagram indicating the patients and healthy individuals included in the study. np, number of patients; ns, number of samples. (DOCX) [file pntd.0002048.s001.docx]

**STARD flowchart**

Culture-negative

Serologically positive

n_p_=48 n_s_=86

Other

diseases

n_p_=46

Occupational

exposed

n_p_=30

Febrile

syndrome

n_p_=34

Blood donors

n_p_=240

Culture-positive

n_p_=25 n_s_=52

Clinical diagnosis of brucellosis

n_p_=73

Non-brucellosis

n_p_=350

Patients and healthy individuals included in the study

n_p_=423 n_s_=488
