# Supplementary material for: Development and Validation of a Novel Diagnostic Test for Human Brucellosis Using a Glyco-engineered Antigen Coupled to Magnetic Beads
Source: PLoS Negl Trop Dis. 2013 Feb 14;7(2):e2048. doi: 10.1371/journal.pntd.0002048 (PMC3573069; doi:10.1371/journal.pntd.0002048)
Supplement: Table S1 — Diagnostic laboratory findings in culture-positive brucellosis patients. (DOCX) [file pntd.0002048.s002.docx]

**Table S1**. Diagnostic laboratory findings in culture-positive brucellosis patients.

| **Patient N°** | **Samples^a^** | **Result of serological tests^b^** | | | | | | ***Brucella sp*. isolated^c^** | **Glyco-assay (%)^d^** | **Clinical symptoms and signs^e^** | |
| --- | --- | --- | --- | --- | --- | --- | --- | --- | --- | --- | --- |
|  |  | **RBT** | **SAT** | **TAT** | **2ME** | **CELISA** | **CFT** |  |  | **Systemic** | **Focal** |
| 1 | a(0) | POS | >200 | 1600 | 400 | 76 | 1280 | *B. suis* bv 1  (0) | 79.3 | Yes | No |
|  | b(2) | POS | >200 | 400 | 200 | 76 | 640 |  | 131.0 | No | No |
|  | c(5) | POS | >200 | 3200 | 800 | 94 | 1280 |  | 255.9 | No | No |
|  | d(9) | POS | >200 | 800 | 800 | 91 | 640 |  | 296.9 | No | Yes |
|  | e(13) | POS | >200 | 200 | 100 | 92 | 320 |  | 223.3 | No | Yes |
|  | f(17) | POS | >200 | 200 | 200 | 81 | 160 |  | 201.1 | No | Yes |
|  | g(20) | POS | >200 | >400 | >100 | 55 | 80 |  | 137.0 | No | Yes |
|  | h(23) | POS | 100 | 100 | 200 | 60 | 80 |  | 151.9 | No | No |
|  | i(26) | POS | 200 | 400 | 50 | 73 | 40 |  | 111.8 | No | No |
|  | j(29) | POS | 100 | 100 | 25 | 75 | 40 |  | 122.4 | No | No |
| 2 | a(0) | NEG | 25 | 25 | NEG | 38 | NEG | *B. suis* bv 1  (-5 years) | 18.1 | No | No |
|  | b(3) | NEG | NEG | NEG | NEG | 36 | NEG |  | 17.1 | No | No |
|  | c(7) | NEG | NEG | 25 | NEG | 38 | NEG |  | 22.1 | No | No |
|  | d(9) | NEG | NEG | NEG | NEG | 37 | NEG |  | 19.8 | No | No |
|  | e(12) | NEG | NEG | NEG | NEG | 36 | NEG |  | 17.8 | No | No |
| 3 | a(0) | POS | >200 | 400 | 100 | 63 | 320 | *B. suis* bv 1  (-4 months) | 105.9 | Yes | No |
|  | b(13) | POS | >200 | 400 | 400 | 91 | 160 |  | 302.3 | Yes | No |
|  | c(17) | POS | >200 | 400 | 200 | 89 | 160 |  | 226.9 | Yes | Yes |
|  | d(20) | POS | 100 | 200 | 100 | 85 | 80 |  | 232.1 | ND | ND |
|  | e(30) | POS | 50 | 50 | 25 | 72 | 20 |  | 111.7 | Yes | No |
|  | f(32) | POS | 50 | 25 | NEG | 71 | 20 |  | 126.8 | No | No |
| 4 | a(0) | POS | >200 | 800 | 200 | 77 | 160 | *B. abortus* bv 1  (-4 months) | 73.6 | No | No |
|  | b(3) | POS | 100 | 200 | 25 | 68 | 20 |  | 58.5 | No | No |
| 5 | a(0) | POS | >200 | 200 | 25 | 32 | 5 | *B. melitensis* bv 1  (-2 years) | 24.1 | Yes | Yes |
| 6 | a(0) | POS | >200 | 400 | 100 | 64 | 320 | *B. abortus* bv 1  (0) | 102.2 | Yes | No |
|  | b(3) | POS | 200 | 100 | 25 | 61 | 80 |  | 65.6 | No | Yes |
|  | c(8) | NEG | 50 | 50 | NEG | 48 | 40 |  | 58.5 | No | No |
| 7 | a(0) | POS | >200 | 400 | 200 | 64 | 160 | *B. suis* bv 1  (0) | 121.6 | Yes | No |
|  | b(2) | POS | >200 | 400 | 200 | 74 | 320 |  | 167.4 | No | No |
|  | c(6) | POS | 50 | 50 | 50 | 52 | 40 |  | 74.6 | No | No |
| 8 | a(0) | POS | >200 | 1600 | 400 | 87 | 40 | *B. abortus*  (-4 months) | 96.7 | Yes | Yes |
| 9 | a(0) | POS | 25 | 25 | NEG | 67 | 5 | *B. abortus* bv 1  (0) | 59.2 | No | Yes |
| 10 | a(0) | POS | >200 | 400 | 100 | 65 | 10 | *B. abortus* bv 2  (0) | 61.5 | Yes | No |
|  | b(5) | NEG | 50 | 50 | NEG | 45 | 5 |  | 27.0 | No | No |
|  | c(10) | NEG | 25 | 50 | NEG | 43 | NEG |  | 22.3 | No | No |
|  | d(12) | NEG | 25 | 25 | NEG | 30 | NEG |  | 15.5 | No | No |
| 11 | a(0) | POS | 100 | 25 | 50 | ND | ND | *B. suis* bv 1  (-2 months) | 148.0 | No | No |
| 12 | a(0) | POS | 100 | 50 | NEG | 75 | 10 | *B. abortus* bv 1  (-3 years) | 50.5 | Yes | Yes |
| 13 | a(0) | NEG | 25 | 25 | NEG | 43 | 10 | *B. abortus* bv 1  (-1 year) | 29.3 | No | No |
| 14 | a(0) | POS | 50 | 100 | NEG | 67 | 80 | *B. suis* bv 1  (-3 years) | 78.6 | No | Yes |
| 15 | a(0) | NEG | 25 | 25 | NEG | 44 | 5 | *B. abortus* bv 1  (-2 years) | 24.7 | No | No |
|  | b(3) | NEG | 25 | 50 | NEG | 41 | NEG |  | 23.8 | No | No |
| 16 | a(0) | POS | 50 | 50 | 25 | ND | ND | *B. melitensis*  (0) | 76.9 | Yes | No |
| 17 | a(0) | POS | 1600 | 1600 | 50 | ND | ND | *B. melitensis*  (0) | 76.7 | Yes | No |
| 18 | a(0) | POS | 800 | 400 | 100 | ND | ND | *B. melitensis*  (0) | 64.1 | Yes | No |
| 19 | a(0) | POS | 1600 | 1600 | 100 | ND | ND | *B. melitensis*  (0) | 37.9 | Yes | No |
| 20 | a(0) | POS | 6400 | 6400 | 400 | ND | ND | *B. melitensis*  (0) | 47.2 | Yes | No |
| 21 | a(0) | POS | 800 | 800 | 50 | ND | ND | *B. melitensis*  (0) | 22.7 | Yes | No |
| 22 | a(0) | POS | 50 | 50 | 50 | ND | ND | *B. melitensis*  (0) | 58.7 | Yes | No |
| 23 | a(0) | POS | 100 | 100 | NEG | ND | ND | *B. melitensis*  (0) | 14.2 | Yes | No |
| 24 | a(0) | POS | 3200 | 3200 | NEG | ND | ND | *B. melitensis*  (0) | 73.0 | Yes | No |
| 25 | a(0) | POS | 6400 | 6400 | 50 | ND | ND | *B. melitensis*  (0) | 79.9 | Yes | No |

^a^ 52 serum samples of 25 culture-positive brucellosis patients. The letters indicate consecutive serum samples of patients sampled repeatedly. The numbers in parenthesis indicate the months at which the samples were taken after admission.

^b^ Results of SAT, TAT, TAT-2ME and CFT are shown as titers. For RBT and BPAT, results are indicated as positive (POS) or negative (NEG). For CELISA results are express as % of inhibition. Considered cutoff values: SAT ≥ 25, TAT ≥ 25, CELISA ˃ 28 %I and CFT ≥ 5.

^c^ The numbers in parenthesis indicate the time at which the bacterium was isolated at (0) or before (minus sign) admission.

^d^ Glycoconjugate-magnetic beads assay, results are expressed as percentage of reactivity of the control positive serum.

^e^ The presence or absence of systemic symptoms and signs and/or focal signs at the corresponding sampling time is indicated as Yes or No.

Systemic clinical symptoms and signs: fever, sweats, anorexia, fatigue, weight loss, depression and hepatosplenomegaly. Focal clinical signs: sacroilitiis, peripheral joint arthritis, spondylitis, osteomyelitis, bursitis, synovitis and abscesses.

ND; no data.
