# Supplementary material for: Development and Validation of a Novel Diagnostic Test for Human Brucellosis Using a Glyco-engineered Antigen Coupled to Magnetic Beads
Source: PLoS Negl Trop Dis. 2013 Feb 14;7(2):e2048. doi: 10.1371/journal.pntd.0002048 (PMC3573069; doi:10.1371/journal.pntd.0002048)
Supplement: Table S2 — Serological results in culture-negative, serologically positive brucellosis patients with clinical diagnosis of brucellosis. (DOCX) [file pntd.0002048.s003.docx]

**Table S2**. Serological results in culture-negative, serologically positive brucellosis patients with clinical diagnosis of brucellosis.

| **Patient N°** | **Samples^a^** | **Result of serological tests^b^** | | | | | | **Glyco-assay (%)^c^** | **Clinical symptoms**  **and signs^d^** | |
| --- | --- | --- | --- | --- | --- | --- | --- | --- | --- | --- |
|  |  | **RBT** | **SAT** | **TAT** | **2ME** | **CELISA** | **CFT** |  | **Systemic** | **Focal** |
| 26 | a(0) | POS | >200 | 400 | ND | 52 | 320 | 100.0 | No | Yes |
| 27 | a(0) | POS | 50 | 100 | NEG | 38 | 10 | 23.8 | Yes | No |
|  | b(7) | NEG | NEG | 100 | NEG | 42 | 10 | 27.4 | No | No |
| 28 | a(0) | POS | >200 | 1600 | 400 | 81 | 320 | 155.1 | Yes | No |
|  | b(2) | POS | >200 | 800 | 100 | 66 | 80 | 85.6 | No | No |
|  | c(4) | POS | >200 | 400 | 100 | 48 | 40 | 50.7 | No | No |
|  | d(7) | POS | >200 | 800 | 100 | 46 | 40 | 26.8 | No | No |
| 29 | a(0) | POS | >200 | 3200 | 400 | 78 | 320 | 119.1 | Yes | No |
| 30 | a(0) | POS | 100 | 25 | 25 | 34 | 10 | 32.1 | No | Yes |
| 31 | a(0) | NEG | 25 | 25 | NEG | 54 | 10 | 80.7 | Yes | No |
| 32 | a(0) | POS | >200 | 800 | 100 | 55 | 80 | 56.0 | Yes | Yes |
| 33 | a(0) | POS | 100 | 25 | NEG | 85 | 20 | 108.7 | Yes | No |
| 34 | a(0) | POS | >200 | 800 | 400 | 86 | 640 | 75.9 | Yes | Yes |
| 35 | a(0) | POS | >200 | 100 | 25 | 59 | 80 | 77.8 | Yes | No |
| 36 | a(0) | POS | 100 | 200 | 100 | 74 | 160 | 113.4 | Yes | No |
|  | b(7) | NEG | 50 | 50 | 25 | 56 | 20 | 41.5 | No | No |
|  | c(11) | NEG | 50 | 25 | NEG | 45 | 5 | 51.1 | No | No |
|  | d(16) | NEG | NEG | 25 | NEG | 41 | 5 | 17.9 | No | No |
|  | e(19) | NEG | NEG | 25 | NEG | 42 | 5 | 40.2 | No | No |
|  | f(23) | NEG | NEG | NEG | NEG | 40 | 5 | 35.6 | No | No |
| 37 | a(0) | POS | >200 | 400 | 400 | 68 | 80 | 129.2 | Yes | No |
|  | b(2) | POS | >200 | 400 | 200 | 48 | 80 | 77.1 | Yes | No |
|  | c(3) | POS | >200 | 400 | 100 | 52 | 40 | 82.6 | Yes | No |
|  | d(6) | POS | >200 | 400 | 100 | 62 | 20 | 72.8 | Yes | No |
|  | e(10) | POS | >200 | 200 | 50 | 60 | 20 | 47.0 | Yes | No |
|  | f(32) | POS | 200 | 50 | NEG | 49 | 10 | 24.5 | No | No |
| 38 | a(0) | POS | 50 | 25 | NEG | 45 | NEG | 28.0 | Yes | No |
|  | b(2) | POS | 50 | 50 | NEG | 47 | 20 | 21.5 | Yes | No |
| 39 | a(0) | POS | >200 | 100 | 100 | 88 | 160 | 158.8 | Yes | No |
|  | b(3) | POS | >200 | 800 | 800 | 92 | 320 | 237.5 | Yes | No |
|  | c(4) | POS | >200 | 800 | 400 | 93 | 640 | 249.3 | Yes | Yes |
|  | d(6) | POS | >200 | 200 | 200 | 92 | 320 | 151.2 | No | No |
|  | e(8) | POS | >200 | 100 | 100 | 91 | 80 | 125.9 | Yes | No |
|  | f(10) | POS | 100 | 50 | 25 | 88 | 80 | 80.7 | Yes | No |
|  | g(13) | POS | 100 | 100 | NEG | 82 | 2O | 68.9 | No | No |
|  | h(20) | NEG | 50 | 50 | NEG | 75 | 5 | 41.1 | Yes | No |
|  | i(31) | NEG | 25 | 50 | NEG | 65 | 5 | 25.4 | No | No |
| 40 | a(0) | POS | 100 | 100 | 50 | 53 | 10 | 33.8 | Yes | No |
| 41 | a(0) | POS | 100 | 100 | NEG | 55 | 10 | 36.4 | Yes | No |
| 42 | a(0) | POS | >200 | 400 | 400 | 91 | 1280 | 168.2 | Yes | No |
| 43 | a(0) | POS | 50 | 100 | NEG | 46 | 10 | 35.8 | Yes | No |
| 44 | a(0) | POS | 50 | 50 | NEG | 67 | 10 | 32.4 | Yes | No |
| 45 | a(0) | POS | >200 | 800 | 200 | 88 | 160 | 149.7 | Yes | No |
|  | b(7) | POS | 100 | 50 | 25 | 47 | ND | 51.0 | Yes | No |
| 46 | a(0) | POS | 50 | NEG | NEG | 76 | 40 | 18.3 | Yes | Yes |
| 47 | a(0) | POS | 50 | 50 | NEG | 70 | 20 | 70.9 | No | Yes |
| 48 | a(0) | POS | >400 | 12800 | 1600 | 93 | 2560 | 177.2 | Yes | Yes |
| 49 | a(0) | POS | 100 | 100 | NEG | 59 | 5 | 37.6 | Yes | No |
| 50 | a(0) | POS | 100 | 100 | 25 | 37 | 5 | 21.1 | Yes | No |
| 51 | a(0) | NEG | 25 | 25 | NEG | 45 | 5 | 50.9 | Yes | No |
| 52 | a(0) | NEG | 25 | 25 | NEG | 68 | 10 | 34.0 | No | Yes |
|  | b(1) | NEG | 50 | 50 | NEG | 48 | 10 | 33.9 | No | Yes |
|  | c(8) | NEG | NEG | NEG | NEG | 45 | NEG | 33.4 | No | No |
|  | d(16) | NEG | 50 | 25 | NEG | 49 | 5 | 29.0 | No | No |
|  | e(19) | NEG | 25 | 25 | NEG | 50 | 5 | 29.7 | No | No |
| 53 | a(0) | NEG | 25 | 25 | NEG | 54 | 10 | 40.8 | Yes | No |
| 54 | a(0) | POS | 100 | 50 | 25 | 48 | NEG | 17.0 | Yes | Yes |
| 55 | a(0) | NEG | 50 | 50 | NEG | 42 | 5 | 26.7 | Yes | No |
|  | b(4) | NEG | 50 | NEG | NEG | 30 | NEG | 44.0 | No | No |
| 56 | a(0) | NEG | 25 | 25 | NEG | 56 | NEG | 35.7 | Yes | No |
|  | b(5) | POS | 100 | 50 | NEG | 50 | NEG | 42.9 | Yes | No |
|  | c(6) | POS | 50 | 25 | NEG | 55 | NEG | 34.6 | No | No |
| 57 | a(0) | NEG | 50 | NEG | NEG | 59 | 10 | 40.8 | Yes | No |
|  | b(3) | NEG | 25 | 25 | NEG | 58 | 10 | 45.4 | No | No |
| 58 | a(0) | NEG | 25 | 25 | NEG | 25 | NEG | 16.7 | No | Yes |
| 59 | a(0) | NEG | 100 | 25 | NEG | 35 | 10 | 13.3 | No | No |
| 60 | a(0) | NEG | 50 | 25 | NEG | 59 | 10 | 17.9 | Yes | No |
|  | b(8) | NEG | 50 | 25 | NEG | 45 | NEG | 14.5 | No | No |
| 61 | a(0) | NEG | NEG | NEG | NEG | 32 | 20 | 15.3 | Yes | No |
| 62 | a(0) | NEG | 25 | 25 | NEG | 34 | 5 | 17.0 | No | Yes |
| 63 | a(0) | NEG | 100 | 50 | NEG | 29 | NEG | 17.3 | Yes | No |
|  | b(6) | POS | 50 | 100 | NEG | 28 | NEG | 15.2 | No | No |
| 64 | a(0) | NEG | 25 | 25 | NEG | 54 | NEG | 18.2 | No | Yes |
|  | b(6) | NEG | NEG | NEG | NEG | 56 | NEG | 18.6 | No | Yes |
|  | c(9) | NEG | 25 | 25 | NEG | 57 | NEG | 15.5 | No | Yes |
|  | d(12) | NEG | 25 | NEG | NEG | 59 | NEG | 15.0 | No | Yes |
| 65 | a(0) | NEG | 50 | 25 | NEG | 41 | 5 | 21.0 | No | Yes |
| 66 | a(0) | NEG | 25 | 25 | NEG | 34 | NEG | 15.6 | No | Yes |
| 67 | a(0) | NEG | NEG | NEG | NEG | 56 | NEG | 25.6 | No | Yes |
| 68 | a(0) | NEG | NEG | NEG | NEG | 37 | 5 | 57.4 | Yes | No |
| 69 | a(0) | NEG | 25 | 25 | NEG | 32 | ND | 26.5 | Yes | No |
| 70 | a(0) | NEG | NEG | 25 | NEG | 48 | NEG | 21.6 | Yes | Yes |
|  | b(3) | NEG | NEG | 25 | NEG | 47 | NEG | 40.7 | Yes | Yes |
| 71 | a(0) | NEG | NEG | 25 | NEG | 48 | NEG | 18.2 | No | Yes |
| 72 | a(0) | NEG | NEG | NEG | NEG | 30 | NEG | 18.0 | No | Yes |
| 73 | a(0) | NEG | 25 | NEG | NEG | 32 | NEG | 35.7 | No | Yes |

^a^ 86 serum samples of 48 culture-negative serologically-positive brucellosis patients with clinical diagnosis of brucellosis. The letters indicate consecutive serum samples of patients sampled repeatedly. The numbers in parenthesis indicate the months at which the samples were taken after admission.

^b^ Results of SAT, TAT, TAT-2ME and CFT are shown as titers. For RBT and BPAT, results are indicated as positive (POS) or negative (NEG). For CELISA results are express as % of inhibition. Considered cutoff values: SAT ≥ 25, TAT ≥ 25, CELISA ˃ 28 %I and CFT ≥ 5.

^c^ Glycoconjugate-magnetic beads assay, results are expressed as percentage of reactivity of the control positive serum.

^d^ The presence or absence of systemic symptoms and signs and/or focal signs at the corresponding sampling time is indicated as Yes or No.

Systemic clinical symptoms and signs: fever, sweats, anorexia, fatigue, weight loss, depression and hepatosplenomegaly. Focal clinical signs: sacroilitiis, peripheral joint arthritis, spondylitis, osteomyelitis, bursitis, synovitis and abscesses.

ND; no data.
